# Supplementary material for: In Situ Photopolymerization of Acrylamide Hydrogel to Coat Cellulose Acetate Nanofibers for Drug Delivery System
Source: Polymers (Basel). 2021 Jun 3;13(11):1863. doi: 10.3390/polym13111863 (PMC8200032; doi:10.3390/polym13111863)
Supplement: Supplementary file 1 [file polymers-13-01863-s001.zip › polymers-1235147-supplementary.pdf]

Supporting information

In situ Photopolymerization of Acrylamide Hydrogel to Coat Cellulose Acetate Nanofibers for Drug Delivery System

Mohamed F. Attia[1,2]*, Ahmed Saad[3]*, Md Arifuzzaman[1], Megan Pitz[4], Khouloud Jlassi[5], Angela Alexander-Bryant[4], Stephen S Kelly[6], Frank Alexis[7], Daniel C. Whitehead[1]*

[1] Department of Chemistry, Clemson University, Clemson, SC 29634, USA

[2] Center for Nanotechnology in Drug Delivery and Division of Pharmacoengineering and Molecular Pharmaceutics, Eshelman School of Pharmacy, University of North Carolina at Chapel Hill, NC 27599, USA

[3] Pretreatment and Finishing Department, Textile Research Division, National Research Center, Dokki, 12622 Cairo, Egypt

[4] Department of Bioengineering, Clemson University, Clemson, SC 29634, USA

[5] Center for Advanced Materials, Qatar University, P. O. Box 2713, Doha, Qatar

[6] Department of Forest Biomaterials, College of Natural Resources, North Carolina State University, Raleigh, NC 27695-8005, USA

[7] School of Biological Sciences and Engineering, Yachay Tech University, 100650-Urcuquí, Ecuador

Submitted to: ***Polymers* – “a special issue Polymers for Biomedical Imaging and Therapy”**

* Corresponding authors:

Mohamed F. Attia: mattia@email.unc.edu and mattianrc@gmail.com

Daniel C. Whitehead: dwhiteh@clemson.edu

Ahmed S. Montaser: abohabbiba2012@gmail.com and amontas@ncsu.edu

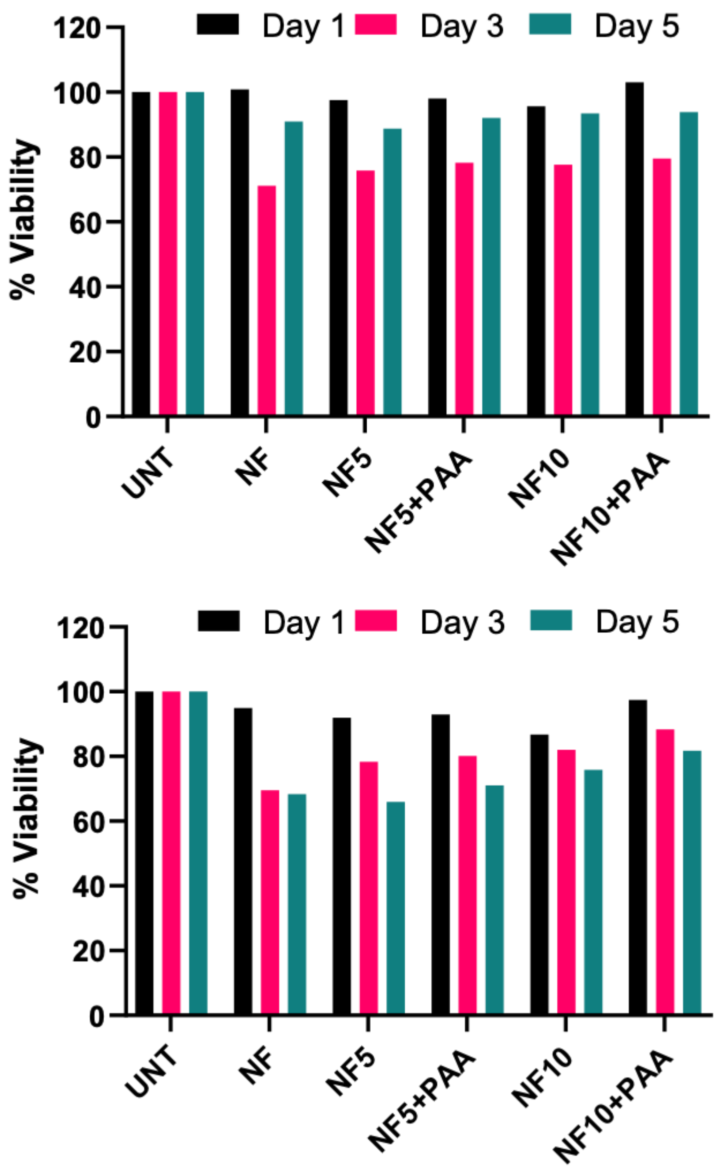

Figure S1. Cytotoxicity assays of the electrospun CANFs. Top: 4 mg/mL; Bottom: 8 mg/mL. UNT denotes untreated 3T3 adipose cells, NF denotes unmodified CANF, NF5 denotes 5 wt. % Ib-loaded CANF, NF5+PAA denotes 5 wt. % Ib-loaded CANF coated with poly-AAm polymer, NF10 denotes 10 wt. % Ib-loaded CANF, and NF10+PAA denotes 10 wt. % Ib-loaded CANF coated with poly-AAm polymer.
